# Chronic liver disease classification using deep learning with SHAP-optimized hybrid features

**DOI:** 10.1016/j.isci.2025.113972

**Published:** 2025-11-06

**Authors:** Naif Almusallam, Salman Khan

**Affiliations:** 1 Department of Management Information Systems, School of Business, King Faisal University, Al Ahsa, Saudi Arabia; 2 Department of Computer Science, Abdul Wali Khan University Mardan, KPK, Pakistan

**Keywords:** health sciences, machine learning

## Abstract

The liver is a vital organ responsible for essential functions, including digestion, metabolism, detoxification, and immunity. Liver disorders, whether due to disease, injury, or congenital conditions, pose serious health risks and require timely diagnosis for effective treatment and improved survival. Advances in machine learning (ML), particularly deep learning, have demonstrated significant potential for disease prediction, offering clinicians more accurate and efficient diagnostic tools. In this study, we propose a novel predictive framework based on a deep neural network (DNN) integrated with feature ranking and projection-based algorithms for accurate liver disease detection. To enhance model interpretability, SHapley Additive exPlanations (SHAP) were applied to identify the most influential features affecting predictions. Experimental results indicate that the proposed DNN model outperforms traditional ML algorithms and state-of-the-art methods, achieving an average accuracy (ACC) of 92.50% under 10-fold cross-validation. These results emphasis its potential to improve diagnostic ACC, support early intervention, and enhance patient outcomes.

## Highlights

• A deep learning model accurately classifies liver disease from benchmark data
• Random oversampling resolves class imbalance and boosts model robustness
• Hybrid features from PCC, GR, RFA, ANOVA, CHI2, PCA, and LDA enhance learning
• SHAP selection improves interpretability and computational efficiency

## Graphical abstract

**Figure:**
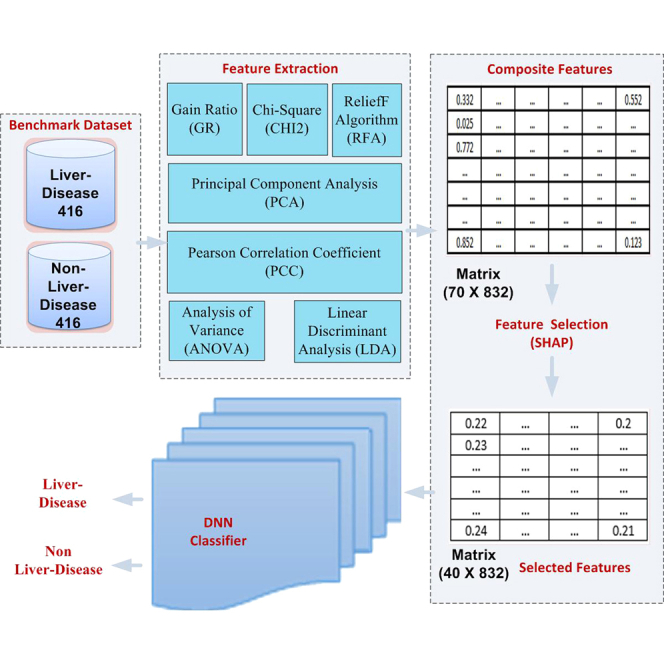

## Introduction

The liver, a vital organ in the human body, plays a crucial role in maintaining most physiological processes that promote health and well-being. It plays an essential role in digestion, metabolism, detoxification, and immune protection, underscoring its importance for internal homeostasis. Despite its regenerative abilities and structure, the liver can easily become infected, physically traumatized, and suffer from inherited problems. A profound impact on its operation, and it may be life-threatening unless appropriate treatment is initiated on time. Even with the liver’s high regenerative capacity, the organ can gradually impair its function and alter the body’s activities with chronic liver injury.^1^^,^^2^ Using efficient means to diagnose and prevent the disease in the early stages can help reduce the effects of liver disease (LD). Understanding the causes and pathological course of liver processes, such as inflammation, fibrosis, and jaundice, is crucial to implementing the necessary medical measures at the right time. Liver disorders, left untreated, can become unmanageable, reducing lifespan and quality of life. Considering that the liver is an essential organ that helps maintain normal health, current medical research focuses on improving the quality of diagnostic methods and procedures, as well as treatment options.^3^^,^^4^ The active detection and management of LD are crucial for reducing the global burden and improving patient outcomes through targeted, timely healthcare interventions.

Over the past several years, the healthcare sector has undergone significant changes, primarily driven by the rapid adoption of data processing technologies, including machine learning (ML) and artificial intelligence (AI).^5^ The technologies have also revolutionized data gathering, preservation, and analysis, enabling healthcare companies to incorporate high-end decision-support systems into their daily clinical practices and to significantly improve diagnosis, treatment decision-making, and the quality of care for patients. ML and AI have also simplified the identification of symptoms of chronic diseases, such as diabetes, high blood pressure, chronic obstructive pulmonary disease (COPD), and heart disease, at the early stage of their manifestation. The technologies enable the use of extensive medical datasets to generate valuable information, contributing to disease prognosis, risk assessment, and treatment. In addition to being used as a diagnostic tool, AI and ML are transforming various aspects of healthcare, including the development of precision medicine and tailored treatment solutions, as well as more efficient drug-discovery processes. Such technologies not only improve the precision of medical estimates but also reduce healthcare costs and improve clinical outcomes. Remarkably, preventive health strategies, health promotion, and early screening also complement the efficacy of such innovations, as their establishment enhances the positive outcomes of technologically advanced healthcare frameworks. As the technological spheres of AI and ML continue to expand daily, the number of healthcare applications is likewise increasing at an unprecedented pace [12]. Whether it is the superior capacity to oversee patients or the efficiency of their hospital operations, they are creating a future that will be more proactive, personalized, and efficient in the provision of their services. Technological advancements will impact modern medicine, particularly in disease diagnosis and treatment, as well as population health management.

Many other researchers have proposed multiple ML methods to improve the precision and efficiency of LD classification.^6^^,^^7^^,^^8^^,^^9^^,^^10^ For example, Babu et al.^11^ employed a *k-*means clustering framework in 2016 to identify liver disorders. They compared its performance with that of a combination of various classifiers, including Naive Bayes (NB), C4.5 decision trees, and k-nearest neighbor (KNN). Their findings indicated that NB achieved 56% accuracy (ACC), KNN achieved 64% ACC, and C4.5 achieved 69% ACC in performance modeling, where clustering was combined with their approach. Later, Gan et al.^12^ proposed a hybrid model called AdaC-TANBN, which combines AdaBoost with an enhanced tree-augmented Naive Bayes (TANBN). The proposed AdaC-TANBN achieved an ACC of 69.03%, which is higher than that of conventional classifiers, demonstrating the usefulness of combining boosting techniques with probabilistic models in predicting LDs. Further, Anagaw et al.^13^ proposed a model leveraging complement Naive Bayes (CNB) alongside other classifiers, achieving 71.36% ACC and outperforming standard NB models, underscoring CNB’s effectiveness in LD classification. In addition, Sreejith et al.^14^ utilized chaotic multi-verse optimization (CMVO) to select features and employed Synthetic Minority Over-sampling Technique (SMOTE) to address class imbalance, achieving an ACC of 82.62% on the Indian liver patient dataset (ILPD). Additionally, Kuzhippallil et al.^15^ achieved the highest ACC of 88.10% by using CMVO to select features and implementing SMOTE to address class imbalance, resulting in an ACC of 82.62% on the ILPD dataset. Moreover, P. Kumar et al.^16^ proposed the Variable-neighbor weighted fuzzy k-nearest neighbor (Variable-NWFKNN) method. The proposed Variable-NWKNN was applied to three liver function datasets: BUPA, ILPD, and MPRLPD. It achieved accuracies of 73.91%, 77.59%, and 87.01%, respectively. After applying the TL_RUS preprocessing technique, the accuracies increased to 78.46%, 87.71%, and 95.79%, highlighting its effectiveness in improving the performance of imbalanced LD datasets. Recently, Amin et al.^17^ achieved the highest ACC of 88.10% using a projection-based statistical technique combined with classifiers, including support vector machine (SVM), logistic regression (LR), and random forests (RFs), thereby significantly improving LD prediction ACC.

The study proposes an accurate and reliable learning model that effectively differentiates LD. First, the effects of overfitting and class imbalance during model development are mitigated by a random oversampling technique that enhances model training. Second, we use two feature processing approaches: feature ranking and statistical projection. The feature ranking algorithms include Pearson correlation coefficient (PCC), gain ratio (GR), ReliefF algorithm (RFA), analysis of variance (ANOVA), and the chi-square (CHI2). The statistical algorithm includes principal-component analysis (PCA) and linear discriminant analysis (LDA). These seven feature sets are then concatenated to form a hybrid feature vector. The SHAP^18^ technique is employed to reduce the feature space, thereby maximizing computational efficiency and removing noisy and redundant features. Using this interpretable technique, the most effective features are highlighted and their effects on the model’s predictions are established. Finally, a multilayer deep neural network (DNN) algorithm is applied as a classifier to build the proposed computational model. To analyze the proposed model’s performance, we conduct rigorous 10-fold cross-validation (CV) on a benchmark dataset. The experimental results exhibit that the proposed predictor produced an average ACC of 92.50%. Moreover, the proposed model’s performance is compared with that of existing models, and the results show that it outperforms them in terms of ACC and other performance metrics.

## Results

### Benchmark dataset

The dataset used in this research is the ILPD,^17^ which comprises 583 data records and contains the 10 most important characteristics related to liver functioning. Table 1 provides detailed descriptions of the attributes of the ILPD dataset. In the data, there is a substantial inequality in the gender category, with 439 men and 144 women (in percentage terms, this corresponds to 75.30% and 24.70%, respectively). The purpose of the dataset classification is to detect LD, grouping individuals based on whether they have it.

**Table 1:** Reordered description of features in the ILPD dataset

Feature	Description
Age (years)	age of individuals (ranging from 4 to 90 years)
Alanine aminotransferase (SGPT)	level of alanine aminotransferase
Total protein (TP)	total protein content in the blood
Albumin and globulin ratio (AGR)	the ratio between albumin and globulin levels
Direct bilirubin (DB)	measurement of direct bilirubin
Gender	participant’s gender
Albumin (ALB)	level of albumin in the blood
Alkaline phosphatase (ALP)	the level of alkaline phosphatase enzyme
Total bilirubin (TB)	total bilirubin concentration
Aspartate aminotransferase (SGOT)	enzyme levels related to liver function

A significant way to predict the long-term risk of LD is through a binary classification task to differentiate between cases (LD and non-LD). The original dataset (ILPD) contains 416 LD-labeled instances and 167 non-LD instances. To address the issue of class imbalance, which has a detrimental impact on the performance of ML models, the random oversampling approach was employed.^17^ This statistical procedure was used to create an equal dataset of 832 samples, with 416 samples in each class (LD and non-LD). To ensure that features with different value ranges contribute equally during model training, all numerical attributes were rescaled using Min-Max normalization.^19^ This technique normalizes feature values to the [0, 1] range, thereby reducing bias toward larger values and improving model convergence and performance. To gain a deeper understanding of the dataset, we conducted a more detailed statistical analysis of its numerical characteristics. Table 2 presents the essential statistical characteristics—minimum, maximum, mean, and standard deviation—for each feature. This analysis helps understand the dispersion and variance of information across attributes, which is required during preprocessing and model training.

**Table 2:** Statistical properties of features in the balanced dataset

Feature	Max	Mean	± SD	Min
TP	9.6	6.5	±1.02	2.7
ALP	2110	267.26	±212.62	63
AGR	2.8	0.98	±0.30	0.3
SGOT	4929	88.78	±245.07	10
DB	19.7	1.16	±2.42	0.1
SGPT	2000	66.78	±155.16	10
TB	75	2.65	±5.32	0.4
Age	90	43.55	±16.28	4.0
ALB	5.5	3.19	±0.76	0.9

Furthermore, we conduct independent validations to represent real-world settings better and assess the model’s generalizability. The dataset, sourced from Kaggle, contains medical records of LD cases and incorporates biochemical and demographic features from real-world clinical data. We obtained a dataset comprising 500 records with 10 features (200 positive and 300 negative) for imbalance-independent validation, which better reflects the model’s generalizability. Importantly, the absence of repeated samples from the training data in the test set does not guarantee an objective assessment of the model’s performance.

### Performance metrics

To elaborate on the analysis of the proposed models’ performance, key assessment indicators were implemented, i.e., sensitivity (SN), specificity (SP), ACC, and the Matthews correlation coefficient (MCC).^20^ Such measures enable a comprehensive understanding of the model’s predictive capacity in various aspects. ACC is the proportion of correctly classified instances to the total number of samples, a general measure of a model’s performance. SN, also known as recall, is the proportion of positives the model correctly predicts; it represents the percentage of patients with LD who are correctly identified as having it. On the other hand, SP is the number of true negatives correctly diagnosed, i.e., the model’s ability to identify cases of non-disease. Lastly, MCC provides a balanced overall measure of all four components of the confusion matrix; thus, it is especially suitable in cases where the dataset is unbalanced, meaning that one or more classes are rare. Together, the metrics have provided a convenient theoretical framework for assessing the ACC and predictive power of LD classification. The following are the corresponding formulas of the calculations.

(Equation 1) $$\text{ACC}=\frac{\text{TP}+\text{TN}}{\text{TN}+\text{TP}+\text{FN}+\text{FP}}\text{.}$$

(Equation 2) $$\text{SP}=\frac{\text{TN}}{\text{TN}+\text{FP}}\text{.}$$

(Equation 3) $$\text{SN}=\frac{\text{TP}}{\text{TP}+\text{FN}}\text{.}$$

(Equation 4) $$\text{MCC}=\frac{\left(\text{TP}\;X\;\text{TN}\right)-\left(\text{FP}\;X\;\text{FN}\right)}{\sqrt{\left(\text{TP}+\text{FP}\right)\;X\;\left(\text{TP}+\text{FN}\right)\;X\;\left(\text{TN}+\text{FP}\right)\;X\;\left(\text{TN}+\text{FN}\right)\;}}\text{.}$$

Here, TP (true positive) and FN (false negative) refer to the correctly and incorrectly classified cases of LD, respectively. TP means that the model correctly detected a sample with LD; FN implies that the model missed a case of LD. On the other hand, TN (true negative) and FP (false positive) refer to non-LD. TN indicates the actual negative samples in non-LD cases, and FP shows the samples in which the model falsely determined them to be non-LD. All four of these elements form the foundation of the confusion matrix and are essential for assessing measures such as precision and ACC, SN, SP, and MCC.

### Feature selection algorithm

Selection of features is a crucial stage in the ML workflow, as it identifies the most significant features, improves model ACC, reduces bias, and reduces computational complexity.^21^ In this study, SHAP have been applied as a feature selection procedure. SHAP is grounded in cooperative game theory and uses scores of feature importance based on the contribution to model predictions. A clear understanding of the dataset’s structure and size facilitates data splitting and feature selection. Well-formed data enhances efficiency in manipulation, making the predictive model more interpretable and practical in performance. In a more formal sense, SHAP estimates the importance of every feature as shown in Equation 5. In this case, *ϕ*_*i*_ represents the SHAP value of feature *i*, *F* is the entire set of features, and *S* is the subset of features excluding feature *i*. The prediction of the model based on features in a subset *S* is given as the value, i.e., *f*_*S*_(*x*_*S*_), and the prediction with the addition of feature *i* is given as *f*_*S*∪{*i*}_(*x*_*S*∪{*i*}_). The data composition and magnitude of the dataset are fundamental to understanding appropriate data preparation, which shapes preprocessing actions such as data segmentation, regularization, and feature selection. A properly organized dataset makes it easy to compute and enables the building of meaningful, robust models.

(Equation 5) $$\text{SHA}P_{i}\left(x\right)=\phi_{i}=\sum_{s\subseteq N\setminus \left\{i\right\}}\frac{\left|S\right|\left(\left|N\right|-\left|S\right|-1\right)}{\left|N\right|}\left[f\left(S\cup \left\{i\right\}\right)-f\left(S\right)\right]\text{.}$$

**Figure 1:**
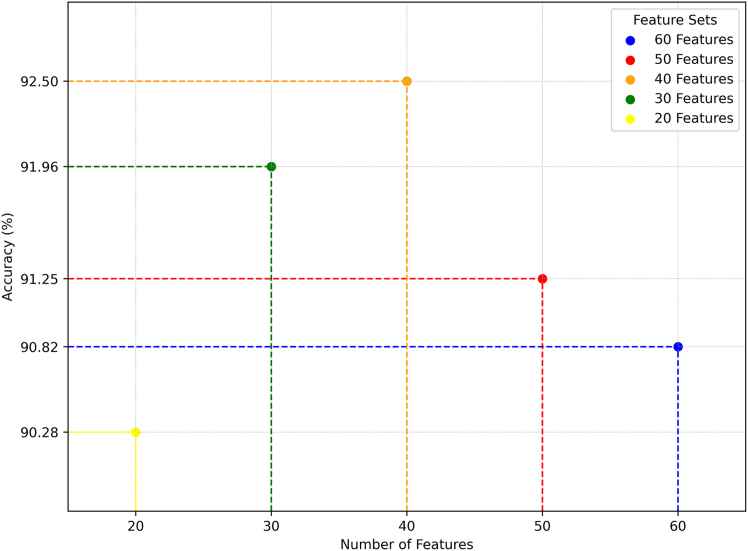
Feature selection using SHAP SHAP was applied to reduce the original 70-dimensional feature set, identifying the most informative features. Model performance was evaluated at each step, with optimal ACC achieved with 40 features; reductions beyond this point led to lower predictive performance.

In this study, we employed SHAP to reduce the original 70-dimensional feature space while preserving the most informative components. We gradually applied SHAP, starting with the complete feature set and incrementally reducing the dimensionality, evaluating model performance at each step, as shown in Figure 1. Different feature subsets were used for experimentation, and based on the results, it was determined that selecting the top 40 features resulted in a noticeable improvement in the overall performance of the proposed prediction model. Further reduction beyond this point led to a noticeable decline in prediction ACC.

**Figure 2:**
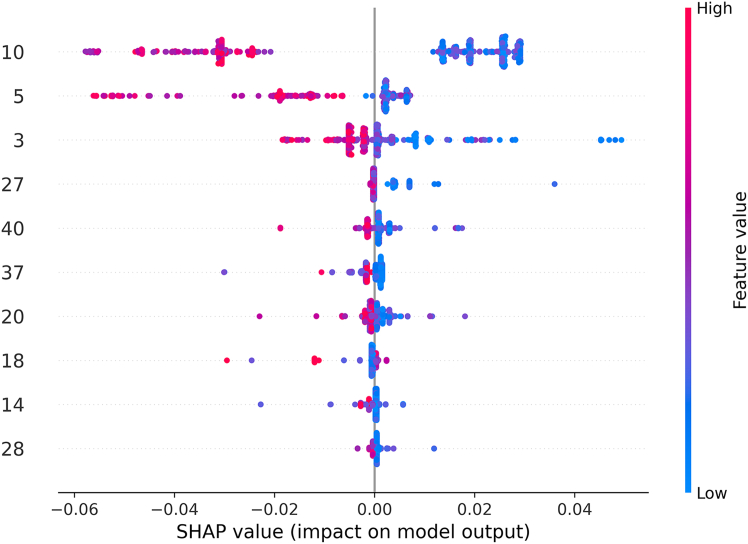
Top 10 features from the optimized 40-dimensional hybrid vector using SHAP The features are ranked by their importance values, highlighting those that most strongly influence the model’s predictions. This visualization emphasizes the key contributors to liver disease classification.

Further, SHAP analysis was applied to identify the most influential features in this study, as shown in Figure 2. Each horizontal row corresponds to a particular feature, and dots of different colors represent individual SHAP values. In the SHAP feature importance plot, the red dots (indicating higher feature values) and the blue dots (indicating lower feature values) show the corresponding SHAP impact on the model output. A positive SHAP value indicates that the feature enhances the likelihood of having LD, while a negative value indicates the opposite.

### Optimization of hyperparameters

**Figure 3:**
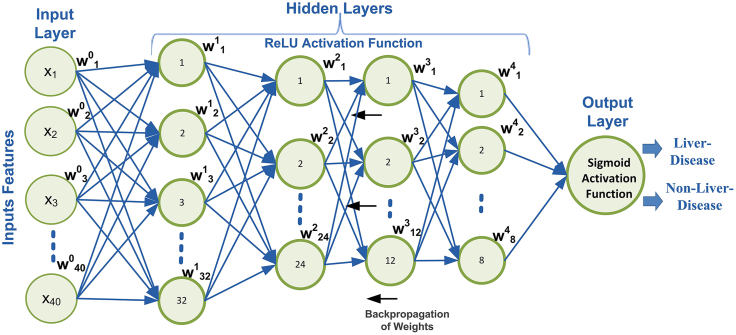
Proposed DNN architecture The figure illustrates the structure of the DNN used for liver disease prediction, including input, hidden, and output layers. The network receives the optimized hybrid feature vector, processes it through multiple hidden layers with nonlinear activations, and produces the final classification output.

This section outlines the hyperparameter setup for the DNN architecture. DNNs, inspired by the structure and function of the human brain, consist of interconnected layers that extract complex hierarchical patterns from data through weighted transformations and activation functions.^22^ Their ability to process high-dimensional, unstructured data makes them effective for medical diagnosis tasks.^23^^,^^24^ The architecture of the proposed DNN model is shown in Figure 3. To analyze the results of the utilized DNN data across different settings, a grid search method was employed.^25^^,^^26^ This method systematically explores various combinations of essential hyperparameters that significantly impact behavior. These hyperparameters, including the activation function, learning rate, and number of training epochs, have been reported to influence learning efficiency and prediction ACC.^22^^,^^24^^,^^26^^,^^27^ The optimal parameter values for the proposed DNN model are presented in Table 3 and were determined through experimentation over a wide range of parameter values.

**Table 3:** Optimized hyperparameters for DNN configuration

Parameter	Optimized value
Momentum	0.9
Weight initialization	Xavier function
Input layer neurons	40
Hidden layer neurons	32–24 – 12–8
Output layer neurons	2
Dense layers	4
Activation functions—input layer	ReLU
Activation functions—output layer	Sigmoid
Learning rate (LR)	0.01
Dropout rate	0.25
Updater	ADAGRAD
Epochs	50
Seed	12345L
Regularization L1	0.001
Optimizer	SGD (stochastic gradient descent)

In initial experiments, the impact of activation functions and the learning rate on the DNN model’s performance was assessed using the specified parameters. According to Table 4, the highest classification ACC is achieved by combining the ReLU activation function and setting the learning rate to 0.01, yielding 92.50% ACC. The findings showed that a gradual decrease in the learning rate led to higher ACC, provided it did not exceed a particular threshold. Nevertheless, reducing the rate further, below 0.01, yielded diminishing returns and did not lead to any significant performance improvement. Hence, both ReLU and a learning rate of 0.01 yield the best set of parameters for optimizing the DNN classifier for LD prediction.

**Table 4:** DNN ACC comparison using activation functions and learning rates

Learning rate (LR)	Sigmoid (%)	ReLU (%)	Tanh (%)
0.01	91.23	92.50	88.87
0.02	90.23	91.92	88.32
0.03	90.01	91.37	87.54
0.04	89.80	90.34	87.14
0.05	89.17	89.91	87.03
0.06	89.04	89.02	86.95
0.07	89.96	88.87	86.65
0.08	89.78	88.08	86.23
0.09	89.65	88.03	85.98
0.10	89.22	87.93	85.23

**Figure 4:**
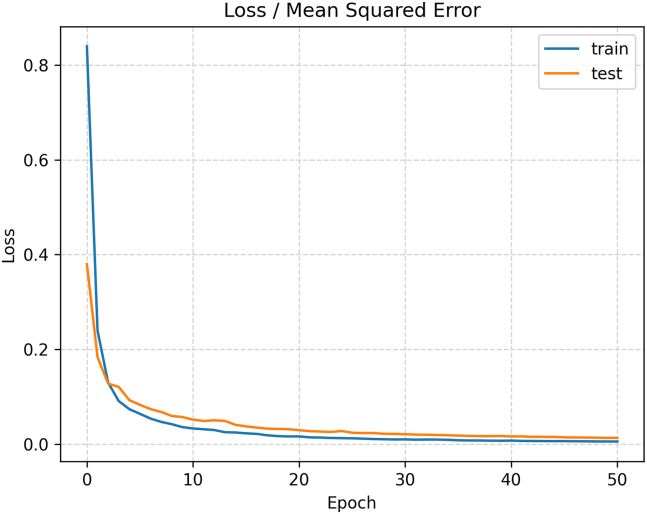
Number of training epochs versus error loss using the ReLU activation function The figure shows the model’s training progression, with error decreasing over successive epochs. It highlights the network’s convergence behavior and the ReLU activation function’s effectiveness in accelerating learning.

Second, the performance of the DNN model was analyzed on different numbers of training epochs. The experimental results are shown in Figure 4. The figures show that error losses are minimized as the number of training epochs increases. For example, the DNN reported an error of 0.892 at the first epoch and continued to minimize, reaching 0.002 after 50 iterations. The figures indicate that 50 epochs are the optimal number of iterations, as the error losses stabilize at this point. Consequently, a set of optimal configurations was obtained through this analysis, and as presented in Table 3.

### Performance analysis

This section presents the analysis of the proposed DNN model’s performance in predicting the use of individual and hybrid feature extraction techniques. To ensure the reliability and generalizability of the assessment, the model was validated using 10-fold CV. However, the integrated feature set may also contain redundant, noisy, and irrelevant information, which could limit its maximum performance. Therefore, we passed the hybrid feature set through the feature selection algorithm to remove noisy, irrelevant, and redundant information. Table 5 summarizes the DNN model’s performance.

**Table 5:** DNN performance with different feature extraction techniques (balanced dataset)

Method	ACC%	SN%	SP%	MCC
PCA	72.37	74.13	70.61	0.481
GR	77.85	79.32	76.38	0.561
RFA	81.32	84.46	78.18	0.665
PCC	82.38	85.64	79.12	0.662
LDA	84.88	88.54	81.22	0.703
CHI2	86.01	87.12	84.91	0.720
ANOVA	86.37	87.79	84.94	0.728
Hybrid feature (before SHAP)	91.76	90.91	92.77	0.835
Hybrid feature (after SHAP)	92.50	93.48	91.43	0.850

As shown in Table 5, the DNN model achieved exceptional performance with a hybrid feature vector, outperforming individual feature extraction methods. To be more precise, the model achieved 91.76% ACC, 90.91% SN, 92.77% SP, and an MCC of 0.835 using the hybrid features. To enhance the model’s predictive power, feature selection was performed using the SHAP method. The DNN also showed improved performance after adding SHAP, achieving 92.50% ACC, 93.48% SN, 91.43% SP, and a higher MCC of 0.850. The results indicate that feature selection is crucial for enhancing the model’s predictive capacity and improving its ACC in detecting LD cases. SHAP integration enhances feature importance and improves classification performance in DL networks.

**Figure 5:**
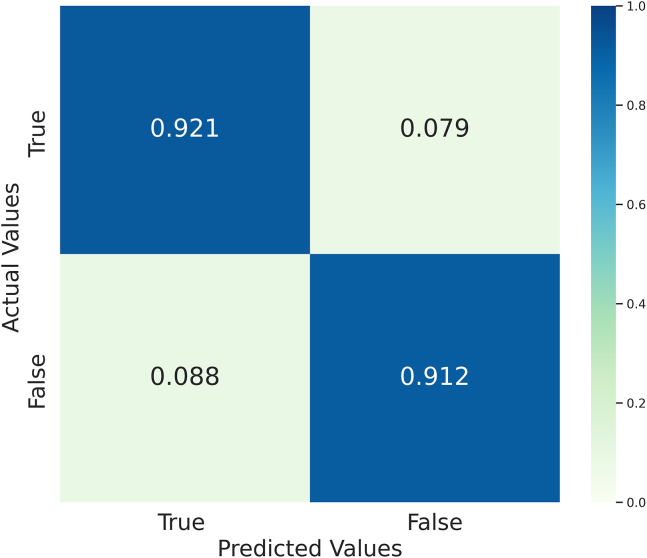
Confusion matrix illustrating the classification performance of the proposed DNN model The matrix shows the number of true positives, true negatives, false positives, and false negatives across all classes. It provides a clear overview of the model’s ACC, precision, and misclassification patterns.

Figure 5 displays the confusion matrix, indicating that the proposed DNN model provides a balanced diagnosis for both positive (LD) and negative (non-LD) cases. This balanced ACC enhances both the model’s ACC and reliability, thereby ensuring its applicability to classification and prediction tasks in diagnostic healthcare applications.

**Figure 6:**
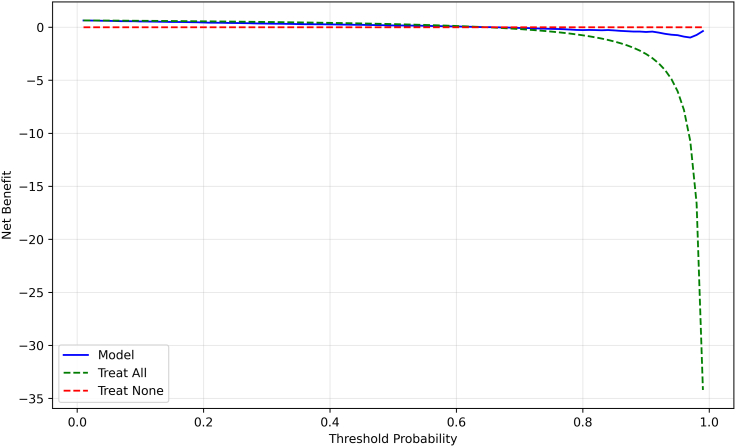
DCA comparing the proposed model with “treat-all” and “treat-none” strategies The curve illustrates the net clinical benefit of the model across different probability thresholds, highlighting its superior decision-making performance compared to baseline strategies.

Moreover, decision curve analysis (DCA) was conducted to assess the clinical utility of the proposed model by evaluating net benefit across varying threshold probabilities, as shown in Figure 6. The DCA compared the model’s performance against “treat-all” and “treat-none” strategies, demonstrating superior decision-making value. This analysis highlights the model’s practical applicability in guiding clinical interventions.

**Figure 7:**
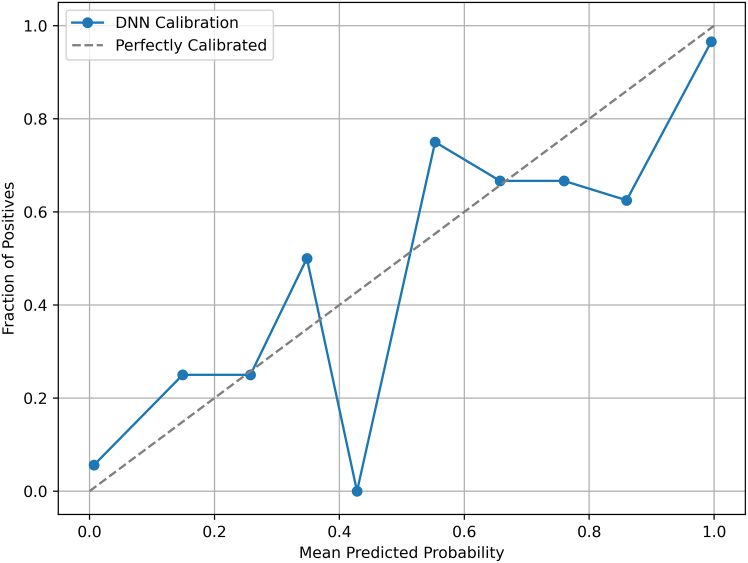
Calibration curve for the proposed DNN model The figure compares the predicted probabilities with the observed outcomes, illustrating the model’s reliability and the agreement between the expected risk and the actual occurrence of liver disease.

Furthermore, Figure 7 illustrates the probabilistic calibration of the proposed DNN model. We computed a calibration curve using scikit-learn’s calibration_curve function with n_bins = 10. The resulting calibration plot compares predicted probabilities against the observed outcome proportions, with the diagonal line representing perfect calibration. Our model closely aligns with the ideal calibration curve, indicating that predicted probabilities are well-calibrated across all bins. The Brier score for the model is 0.0654, which is considered excellent, as values below 0.1 typically reflect well-calibrated models. For comparison, clinical risk prediction models commonly report Brier scores of 0.05–0.20, placing our model at the high-performance end of this spectrum. A lower Brier score indicates superior probabilistic ACC, and our results suggest that the DNN provides reliable probability estimates beyond simple classification ACC. These findings underscore the strong discriminative capability and robust calibration of the proposed approach, both of which are critical for ensuring trustworthy predictions in clinical decision-making.

### Performance comparison with other classifiers

In this section, we compare the performance of the proposed DNN model with commonly used classifiers. These classifiers include RF,^28^ SVM,^29^ LR,^30^ NB,^31^ and KNN.^32^ RF is one of the ensemble methods that builds a set of decision trees from bootstrapped datasets to improve the stability and effectiveness of prediction.^33^ The SVM, which has proven effective for both linear and nonlinear classification problems, particularly in the life sciences, operates by determining the optimal hyperplane that separates the classes.^34^ LR is a widely used statistical model for binary classification, particularly in disease prediction due to its simplicity and interpretability; however, it struggles with complex, nonlinear patterns. NB is a probabilistic classifier based on Bayes’ theorem, offering fast and efficient performance; however, its assumption of feature independence limits ACC in correlated medical datasets. Lastly, the KNNs algorithm is a popular instance-based model that classifies samples based on their distance to the closest neighbors, particularly in image and pattern recognition. Table 6 underlines the comparative efficiency of the above methods on the training data.

**Table 6:** Performance comparison of the proposed DNN model with traditional ML classifiers on the training dataset using 10-fold CV

Classifier	ACC%	SN%	SP%	MCC
Proposed DNN	92.50	93.48	91.43	0.850
SVM	91.83	93.45	90.21	0.819
RF	90.63	92.75	88.52	0.813
LR	89.87	91.12	88.82	0.805
NB	89.83	90.65	89.01	0.796
KNN	89.15	91.02	87.28	0.803

Across all classifiers, the proposed DNN classifier demonstrated superior performance, achieving 92.50% ACC and an MCC of 0.850. The SVM classifier ranked second in overall performance, while the remaining classifiers achieved comparatively competitive but lower results. Notably, the DNN model maintained consistent and stable performance across all evaluation metrics, surpassing the average ACC by 2.24%. This improvement can largely be attributed to the DNN’s multilayered architecture, which effectively captures and models complex, non-linear relationships within the data.

### Comparison with existing state-of-the-art models

In this section, a comparative assessment of the proposed DNN model is presented against existing state-of-the-art models published in the literature. Table 7 presents a comparative analysis of classification ACC among existing approaches on the same benchmark dataset. For instance, Amin et al.^17^ and Kumar et al.^16^ achieved accuracies of 88.10% and 87.71%, respectively, using the RF and Variable-NWFKNN algorithms. In comparison, Altaf et al.^9^ reported 73.56% with a voting classifier. Similarly, Gupta et al.^8^ used LightGBM, achieving an ACC of 63.00%, the lowest among the comparisons. Elias et al.^10^ also employed a voting classifier and achieved a relatively better performance of 80.10%. In contrast, the proposed DNN model outperformed all existing methods, attaining the highest ACC of 92.50%. This result demonstrates the effectiveness of the DNN architecture in modeling complex patterns and improving predictive ACC for LD classification.

**Table 7:** ACC comparison of proposed model with previous state-of-the-art methods

Method	Classifier	ACC%
Gupta et al.^8^	LightGB	63.00
Altaf et al.^9^	Voting	73.56
Elias et al.^10^	Voting	80.10
Kumar et al.^16^	Variable-NWFKNN	87.71
Amin et al.^17^	RF	88.10
Proposed model	DNN	92.50

### Comparison on an independent dataset

Furthermore, we assess the generalization’s ability of our proposed model using separate datasets. Table 8 illustrates the performance of our model. For instance, the proposed model we developed achieved notably superior results, with 90.45% ACC and an MCC of 0.828. In contrast, the SVM predictor attained 89.77% ACC and an MCC of 0.789 on the independent dataset.

**Table 8:** Performance comparison of the proposed DNN model with ML classifiers on an independent dataset

Classifier	ACC%	SN%	SP%	MCC
Proposed DNN	90.45	92.28	88.62	0.828
SVM	89.77	92.31	86.21	0.789
RF	89.39	91.58	87.21	0.781
LR	89.58	92.41	86.76	0.793
NB	88.65	91.46	84.75	0.766
KNN	88.57	91.41	85.73	0.757

## Discussion

LD remains a critical global health challenge, posing a serious threat to patient well-being and necessitating accurate diagnosis for effective treatment. In this study, we presented a robust predictive model based on a DNN for LD classification. To enhance model performance, we addressed the issue of high-dimensional data by applying multiple feature ranking algorithms, including PCC, GR, RFA, ANOVA, and CHI2, to evaluate the importance of individual features related to LD.^35^^,^^36^ The contributions of each feature, as determined by PCC, GR, and RFA, are summarized in Table 9. Statistical projection methods,^37^ such as PCA^38^ and LDA, were also applied to project the original feature space onto the most informative dimensions, improving class separability. SHAP were used to quantify feature importance, providing interpretable insights into each feature’s contribution to the model’s predictions. The outputs of all these techniques were combined into a hybrid feature vector, reducing redundancy while retaining the most relevant information for classification.

**Table 9:** Importance scores of features for liver disease prediction using PCC, GR, and RFA methods

Feature	RFA	PCC	GR
ALB	0.2883	0.1836	0.0408
TP	0.2161	0.0443	0.0000
DB	0.2895	0.3205	0.1421
SGOT	0.2393	0.2017	0.1005
Age	0.2625	0.1596	0.0372
AGR	0.2599	0.2046	0.0822
Gender	0.0250	0.0857	0.0065
SGPT	0.2613	0.2141	0.0701
ALP	0.1936	0.2460	0.0867
TB	0.2848	0.2874	0.1373

Experimental results demonstrated that the proposed DNN model achieved an ACC of 92.50%, outperforming existing methods by over 16% points. This illustrates the model’s ability to capture complex nonlinear relationships and improve predictive performance. Looking ahead, the rapid growth of genomic and biomedical data presents computational challenges for conventional methods.

Looking ahead, the exponential growth of genomic and biomedical data driven by next-generation sequencing technologies poses significant computational challenges for conventional sequential methods. To address these challenges and enhance scalability, future research will focus on implementing parallel computing architectures and leveraging big data analytics platforms. This direction aims to facilitate the efficient processing of large-scale medical datasets, paving the way for the deployment of advanced predictive models in precision medicine and healthcare decision-support systems.

### Limitations of the study

The primary limitation of this study is its reliance on a single benchmark dataset, which may restrict the model’s generalizability to diverse populations and real-world clinical settings. Additionally, the model does not incorporate genomic or multi-omics data, limiting its potential for deeper biological insights into LD mechanisms.

## Resource availability

### Lead contact

Any additional information required to reanalyze the data reported in this paper is available upon request from the lead contact, Salman Khan.

### Materials availability

This study did not generate new unique reagents.

### Data and code availability

• This paper analyzes existing, publicly available data, accessible at https://doi.org/10.24432/C5D02C.
• This paper does not report original code.
• Any additional information required to reanalyze the data reported in this paper is available from the lead contact upon request.

## Acknowledgments

This work was supported by the Deanship of Scientific Research, Vice Presidency for Graduate Studies and Scientific Research, 10.13039/501100020912King Faisal University, Saudi Arabia (grant no. KFU252363).

## Author contributions

All authors contributed equally. Conceptualization, S.K. and N.A.; methodology, S.K.; software and analysis, N.A.; writing – original draft, N.A.; writing – review and editing, S.K.; and supervision, S.K. All authors have read and approved the final version of the manuscript.

## Declaration of interests

The authors declare no conflicts of interests.

## STAR★Methods

### Key resources table

**Table:** 

REAGENT or RESOURCE	SOURCE	IDENTIFIER
**Deposited data**		

The Indian Liver Patient Dataset (ILPD)	https://doi.org/10.24432/C5D02C	C5D02C

### Method details

One of the most common problems with machine learning algorithms is the high dimensionality of data, particularly when a dataset contains a large number of features, and a substantial portion of them are either worthless or redundant.^39^^,^^40^ To address this, we employ five feature-ranking methods—PCC, GR, RFA, ANOVA, and CHI2—alongside projection techniques (PCA and LDA) to identify and extract the most informative liver disease features.

Firstly, the Pearson Correlation Coefficient (PCC), Gain Ratio (GR), and ReliefF Algorithm (RFA) ranking techniques were used to evaluate each feature’s contribution to liver disease classification, as summarized in Table 9. PCC analysis revealed a strong correlation between Total Bilirubin (TB) and Direct Bilirubin (DB) (r = 0.88), with DB showing the highest frequency and relevance. This indicates significant inter-feature relationships and underscores DB’s diagnostic importance among the dataset’s attributes. GR and RFA analyses further confirmed these findings. GR identified DB, TB, and SGOT as the most discriminative features, while RFA highlighted DB, ALP, and TB, ranking gender as the least predictive. Collectively, these results consistently demonstrate the diagnostic significance of DB and TB, providing a strong basis for subsequent feature selection and model refinement.

Secondly, the CHI2 and ANOVA F-score methods were employed to evaluate and rank the importance of features contributing to liver disease prediction. The CHI2 method compared observed and expected frequencies of each feature relative to class labels, effectively identifying attributes with strong discriminative power while reducing redundancy. Meanwhile, the ANOVA F-score ranked continuous features based on their inter-class variance, enhancing the model’s ability to distinguish between disease and non-disease cases. Both methods are computationally efficient and well-suited for large datasets, ensuring that only the most informative features are retained for improved model performance.

Thirdly, Principal Component Analysis (PCA) and Linear Discriminant Analysis (LDA) were applied for dimensionality reduction to enhance the performance and interpretability of the liver disease prediction model. PCA is an unsupervised technique that identifies linearly independent components by analyzing the covariance structure of the data to capture maximum variance. It transforms the original feature set into a smaller set of principal components that retain the most informative patterns, thereby reducing overfitting and computational complexity while preserving essential discriminatory information. LDA, on the other hand, is a supervised method that projects high-dimensional data into a low-dimensional space to maximize class separability. Unlike PCA, which focuses on variance, LDA emphasizes maximizing the ratio of between-class variance to within-class variance, ensuring that data points from the same class are closely grouped while those from different classes are well separated. Together, PCA and LDA complement each other by efficiently reducing data dimensionality and improving the model’s ability to distinguish between liver disease and non-disease cases.

Finally, The results from these methods are integrated into a unified hybrid feature vector by concatenating the individual feature sets derived from Linear Discriminant Analysis (LDA), Principal Component Analysis (PCA), Analysis of Variance (ANOVA), Chi-Square (CHI2), ReliefF Algorithm (RFA), Gain Ratio (GR), and Pearson Correlation Coefficient (PCC). This combined hybrid feature vector, consisting of 70 features, captures the most significant and complementary information for improved prediction ACC.

### Quantification and statistical analysis

All statistical analyses and performance evaluations were conducted using Python (version 3.10) with supporting libraries, including NumPy, pandas, scikit-learn, and TensorFlow. Model performance was quantified using key evaluation metrics, including ACC, Precision, Recall (Sensitivity), Specificity, F1-score, Area Under the Curve (AUC), and Matthews Correlation Coefficient (MCC). These metrics were derived from the confusion matrix to provide a comprehensive assessment of predictive capability and model robustness. Cross-validation (10-fold) was employed to ensure the reliability and generalizability of results. Mean and standard deviation values were reported for all experiments to represent central tendency and dispersion, respectively.
